# Supplementary material for: Aprotinin Inhibits SARS-CoV-2 Replication
Source: Cells. 2020 Oct 30;9(11):2377. doi: 10.3390/cells9112377 (PMC7692688; doi:10.3390/cells9112377)
Supplement: Supplementary file 1 [file cells-09-02377-s001.zip › cells-865472-supplementary/Figure S1.pdf]

Figure S1

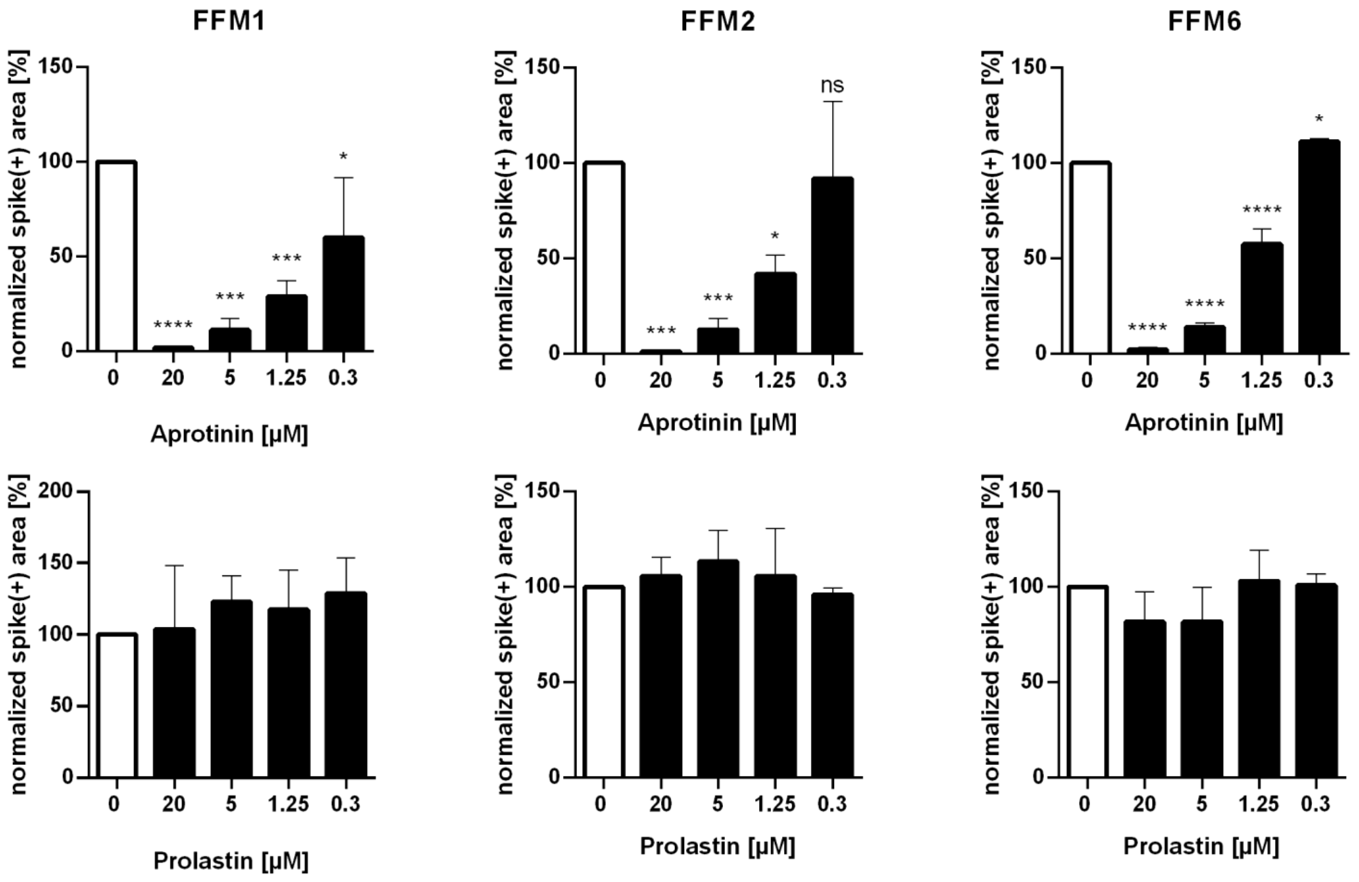

Figure S1. Quantification of immunostaining for the spike protein in SARS-CoV-2-infected (isolates FFM1, FFM2, FFM6) Caco2 cells with and without treatment of aprotinin or SERPINA1/ alpha-1 antitrypsin (prolastin) presented in Figure 2B.
