# Supplementary material for: Aprotinin Inhibits SARS-CoV-2 Replication
Source: Cells. 2020 Oct 30;9(11):2377. doi: 10.3390/cells9112377 (PMC7692688; doi:10.3390/cells9112377)
Supplement: Supplementary file 1 [file cells-09-02377-s001.zip › cells-865472-supplementary/Figure S2.pdf]

Figure S2

Control **PBS**

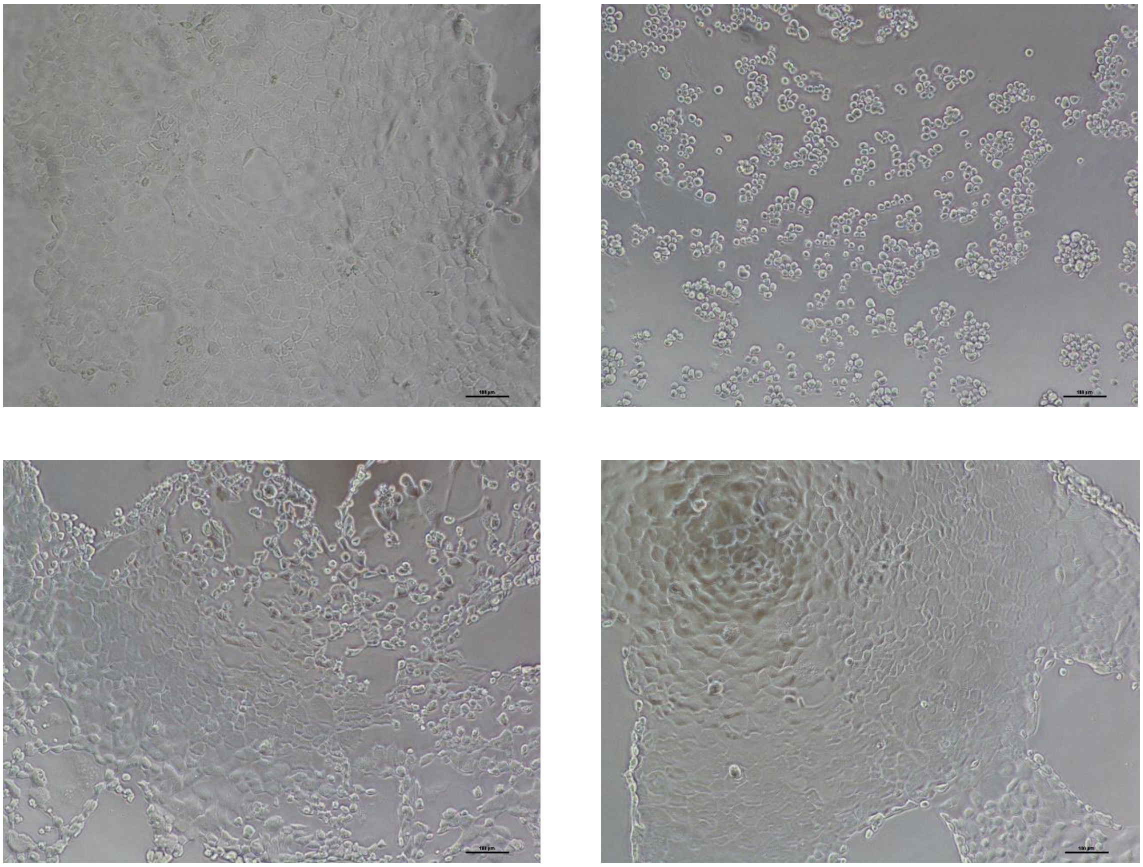

Aprotinin 20µM **SERPINA1 (20µM)**

Figure S2. Trypsin inhibition by aprotinin and SERPINA1/ alpha-1 antitrypsin. Nearly confluent Caco2 cell cultures were washed three times with PBS and incubated with 400µg/ mL trypsin alone or in combination with aprotinin 20µM or SERPINA1 20µM for 2h.

Figure S2

Control PBS

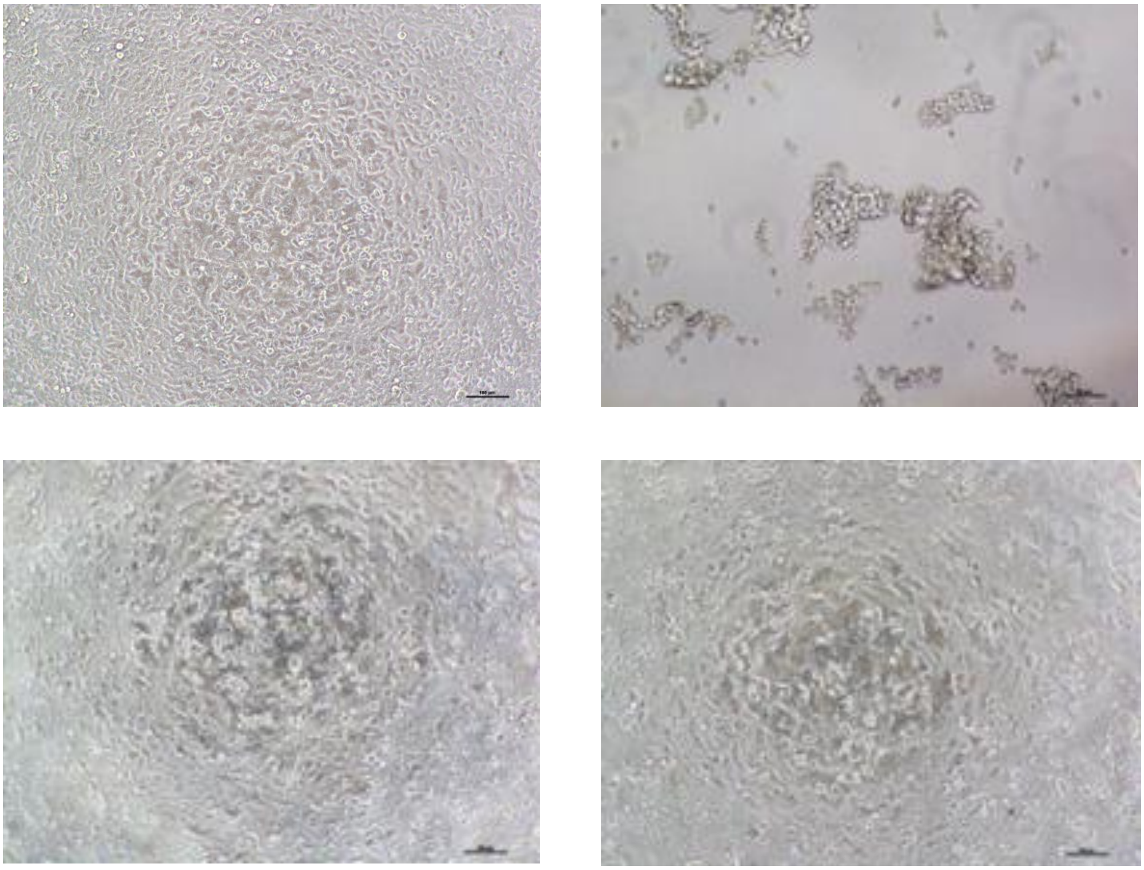

Aprotinin 20µM SERPINA1 (20µM)

Figure S2. Trypsin inhibition by aprotinin and SERPINA1/ alpha-1 antitrypsin. Nearly confluent A549 cell cultures were washed three times with PBS and incubated with 400µg/ mL trypsin alone or in combination with aprotinin 20µM or SERPINA1 20µM for 2h.
