# Supplementary material for: Aprotinin Inhibits SARS-CoV-2 Replication
Source: Cells. 2020 Oct 30;9(11):2377. doi: 10.3390/cells9112377 (PMC7692688; doi:10.3390/cells9112377)
Supplement: Supplementary file 1 [file cells-09-02377-s001.zip › cells-865472-supplementary/Figure S3.pdf]

Figure S3

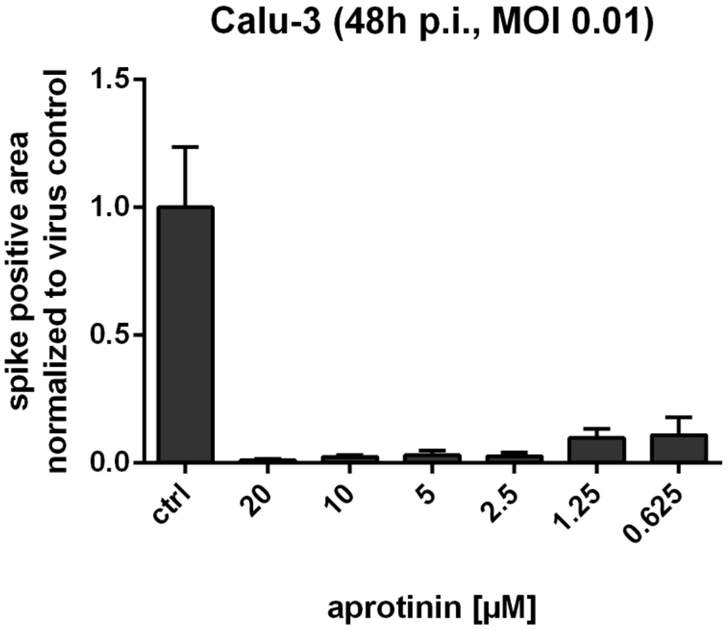

Figure S3. Quantification of immunostaining for the spike protein in SARS-CoV-2/FFM7 (MOI 0.01)-infected Calu-3 cells 48h post infection in response to aprotinin treatment.
