# Supplementary material for: Aprotinin Inhibits SARS-CoV-2 Replication
Source: Cells. 2020 Oct 30;9(11):2377. doi: 10.3390/cells9112377 (PMC7692688; doi:10.3390/cells9112377)
Supplement: Supplementary file 1 [file cells-09-02377-s001.zip › cells-865472-supplementary/Figure S4.pdf]

Figure S4

TMPRSS2

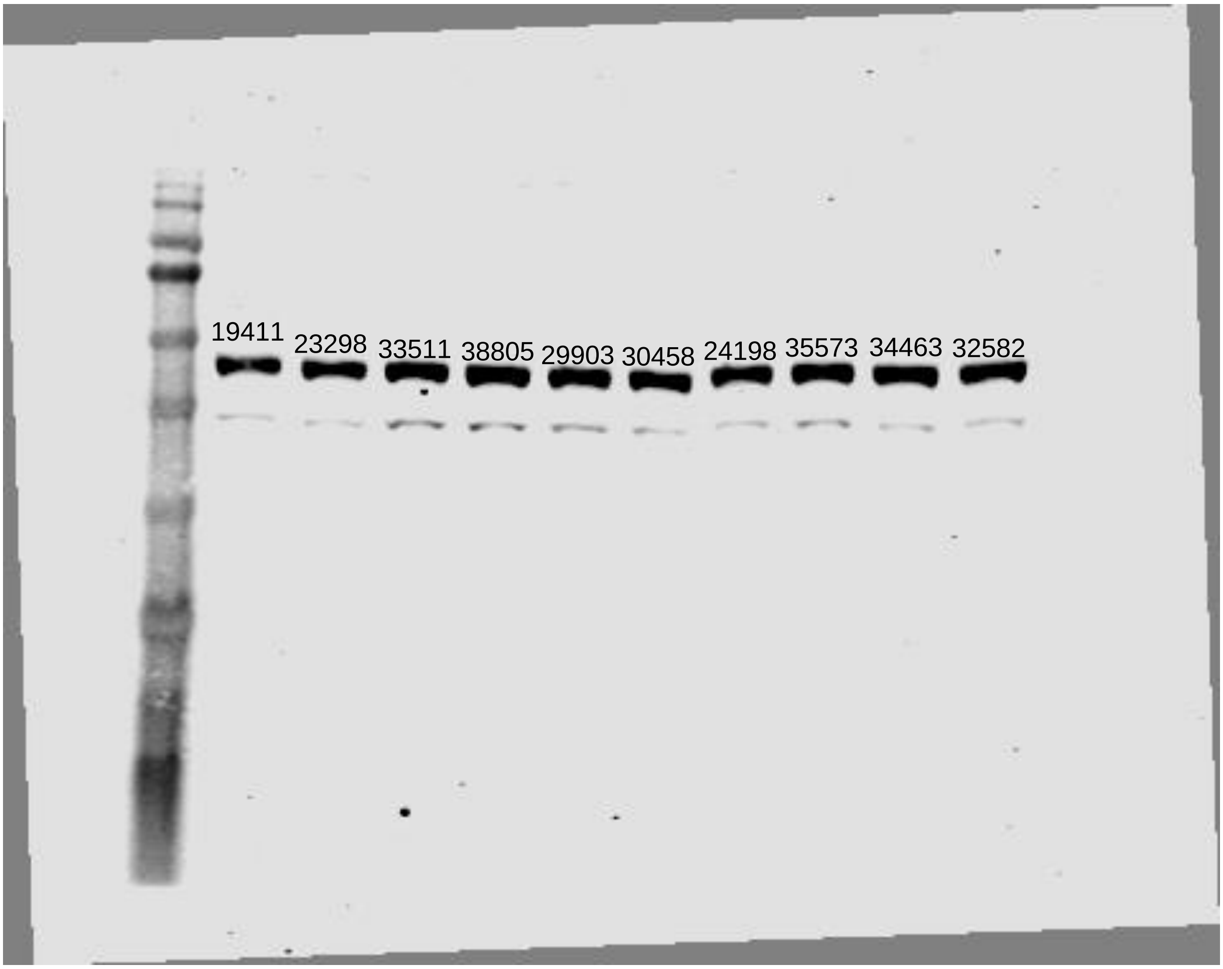

Figure S4

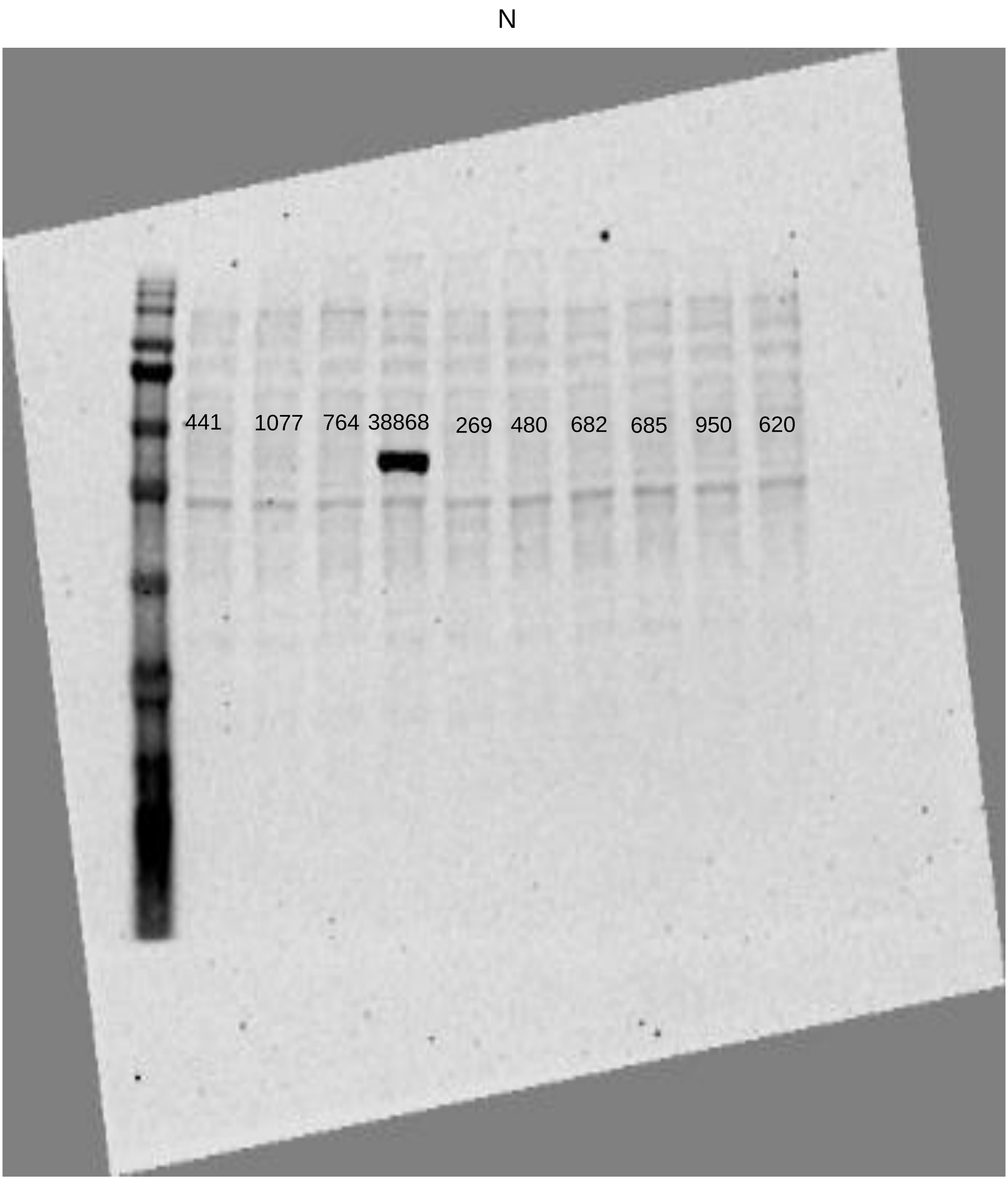

Figure S4

GAPDH

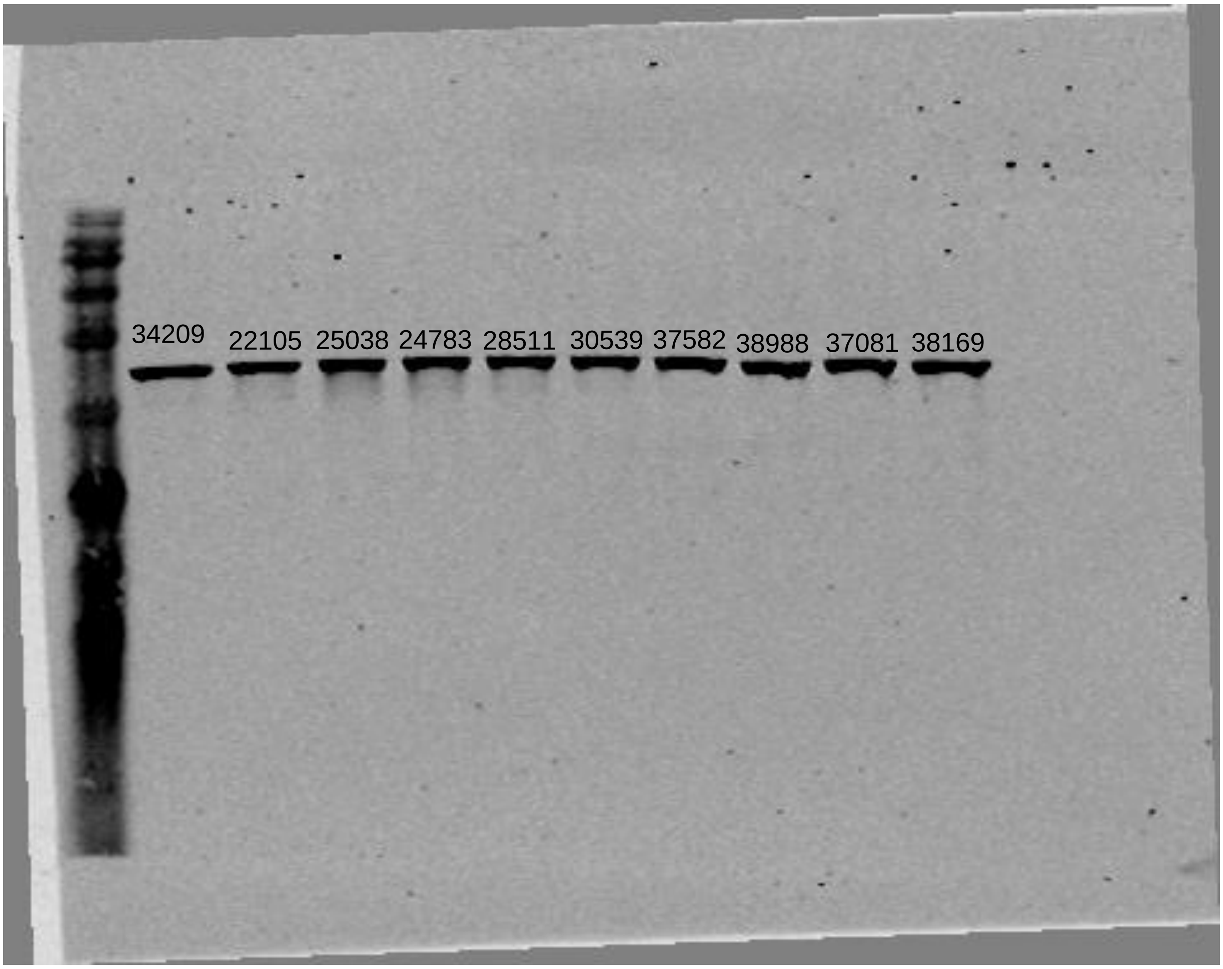

Figure S4. Uncropped Western blots corresponding to Figure 4B. Quantification was performed by laser-induced fluorescence using an infrared scanner (Odyssey, Li-Cor Biosciences) and Image Studio version 3.1 software.
